# Crocin ameliorates MicroRNAs-associated ER stress in type 2 diabetes induced by methylglyoxal

**DOI:** 10.22038/IJBMS.2022.60493.13407

**Published:** 2022-02

**Authors:** Vahid Radmehr, Akram Ahangarpour, Seyyed Ali Mard, Layasadat Khorsandi

**Affiliations:** 1 Student Research Committee, Department of Physiology, Ahvaz Jundishapur University of Medical Sciences, Ahvaz, Iran; 2 Medical Basic Sciences Research Institute, Physiology Research Center, Department of Physiology, School of Medicine, Ahvaz Jundishapur University of Medical Sciences, Ahvaz, Iran; 3 Alimentary Tract Research Center, Ahvaz Jundishapur University of Medical Sciences, Ahvaz, Iran; 4 Department of Anatomical Sciences, School of Medicine, Medical Basic Sciences Research Institute, Cellular and Molecular Research Center, Ahvaz Jundishapur University of Medical Sciences, Ahvaz, Iran

**Keywords:** Crocin, Diabetes, ER stress, Glyoxalase 1, Methylglyoxal, MiR-204

## Abstract

**Objective(s):**

Methylglyoxal (MG) provokes endoplasmic reticulum (ER) stress in β-cells and triggers pancreatic β-cell dysfunction. Crocin has anti-diabetic properties. The present study investigated whether crocin prevented pancreas damages induced by MG.

**Materials and Methods:**

Diabetes was induced by MG administration (600 mg/kg/day, PO). On the fourteenth day, after proving hyperglycemia, crocin (15, 30, and 60 mg/kg) and metformin (MT) (150 mg/kg) were used for detoxification of MG until the end of the experiment. The animals were divided into 6 groups: 1) control, 2) diabetic by MG, 3) MG + crocin 15 mg/kg, 4) MG + crocin 30 mg/kg, 5) MG + crocin 60 mg/kg, and 6) MG + MT. The data were analyzed by one-way analysis of variance and significant differences were compared by Tukey and Bonferroni tests (*P*<0.05). Biochemical assays, antioxidant evaluation, and microRNAs expression associated with ER stress were assessed.

**Results:**

MG induced hyperglycemia, insulin resistance, and dyslipidemia (*P*<0.001). Crocin and MT significantly ameliorated β-cell function through reduction of fasting blood glucose, malondialdehyde levels (*P*<0.001), and significant elevation of anti-oxidant enzyme activity accompanied by regulation of glutathione and glyoxalase1-Nrf2 in MG induced diabetic mice. Crocin and MT significantly down-regulated microRNAs 204, 216b, 192, and 29a expression (*P*<0.001). Crocin (60 mg/kg) (*P*<0.01) and MT (*P*<0.001) could improve diameter of pancreatic islets in MG treated mice.

**Conclusion:**

Crocin prevents the progression of diabetes through modulating ER stress-associated microRNAs and GLO1 activity with the helpful effects of glutathione and Nrf2.

## Introduction

Insulin is responsible for blood sugar regulation (1). This process is mediated by the transfer of glucose from the bloodstream into cells (2). Decreased tissue response to normal insulin action is known as insulin resistance (3). Advanced glycation end products (AGEs) have been demonstrated as one of the factors involved in the progression of insulin resistance. In addition, methylglyoxal (MG) is the main precursor of the AGEs that interacts with its receptors and begins degeneration of cells by an overproduction of ROS that causes pancreatic β-cells damages (4). Also, MG causes toxicity in pancreatic INS-1 cells by mitochondrial dysfunction and finally promotes the ER stress-mediated Ire1-JNK pathway (5). In fact, an imbalance in ROS generation affects insulin secretion and sensitivity, which causes diabetes (6). Beta cells have an extensive endoplasmic reticulum volume for the synthesis and secretion of insulin. Therefore, endoplasmic reticulum dysfunction develops ER stress. RNA-dependent protein kinase-like endoplasmic reticulum kinase (PERK) as a main component of the unfolded protein response (UPR), regulates ER stress and causes ER homeostasis (7). Glyoxalase 1 (GLO1) can promptly clear alpha-carbonyl aldehyde such as MG, with the helpful effect of glutathione (GSH) as a cofactor. Generally, GSH is regulated by nuclear factor erythroid-2-related factor 2 (Nrf2)/anti-oxidant response element pathway (8). The contributing effect of activation of this factor in the face of many diseases has been proven. A study indicated that Nrf2 also can activate GLO1 transcription (9). Also, GLO1 can directly inhibit AGEs formation under the hyperglycemia condition (10). Further, one report showed that an enhancement of GLO1 activity prevented the progression of diabetic nephropathy (11).

MicroRNAs have an important role in identifying physiological processes such as pathogenesis of diabetes (12). MicroRNA-204, a specific biomarker of beta cell loss, directly targets several key pancreatic β-cell genes, including PERK as a sensor of ER stress (13). Also, miR-216b is abundantly expressed in acinar cells and can be a specific biomarker of acinar cell injury (14). It is well-known that miR-29a is associated with insulin secretion in pancreatic β-cells, and prevents the progression of diabetes during ER dysfunction of the β-cells (14). Furthermore, miR-192 is another key biomarker associated with pancreatic β-cell function that regulates βcell proliferation, apoptosis, and inhibition of insulin secretion. In addition, miR-192 suppresses Glucagon-like peptide-1 expression as a strong insulin secretagogue, thereby further promoting type 1 diabetes mellitus (15). 

Metformin is widely used as an antidiabetic drug that diminishes hepatic glucose production and enhances insulin sensitivity. It is proven that some patients do not tolerate metformin, or may develop end-stage diabetes complications such as heart or kidney failure, in which metformin is contraindicated due to the risk of conceiving lactic acidosis (16). The efficacy of plant flavonoids in the control of diabetes has been determined. Crocin is a yellow carotenoid pigment of saffron that exhibits verified pharmacological effects such as anti-oxidant (17), anti-inflammatory (18), and anti-fibrotic **(**19**)** properties. Based on a review, crocin is effective in autoimmune diseases (20). Also, previous studies demonstrated that crocin is an effective compound for treatment of neurodegenerative disease (21, 22). However, no experimental study about the anti-oxidant effects of crocin on MG-induced ER stress through the PERK-Nrf2 signaling pathway has been reported. In the present study, experimental evidence about the anti-oxidant effects of crocin on MG-induced diabetic mice was provided.

## Materials and Methods

***Chemicals and drugs***

MG and crocin were purchased from Sigma (St. Louis, MO, USA). Metformin from Solar, bio (Dangjin, South Korea), Xylazine2% and Ketamine10% from Alfasan Co. (The Netherlands), High Pure RNA Isolation Kit (Roche Diagnostics, Vilvoorde, Belgium), and miScript II RT Kit (QIAGEN, GmbH, Germany) were obtained. Malondialdehyde (MDA), catalase (CAT), superoxide dismutase (SOD), glutathione (GSH), insulin (Monobind Inc, USA), and Nrf2 levels of kidney tissues were all measured by commercial kits (ZellBio GmbH, Germany). In order to assess MG detoxification, GLO1 activity was measured (23).

***Animals***

Male NMRI mice (4 weeks old) weighing 20 ± 5 g were purchased from Ahvaz Jundishapur University of Medical Sciences (AJUMS), Ahvaz, Iran; which is completely attributed by AJUMS animal care guidelines and the study was approved under an animal committee ethics grantee (No. IR.AJUMS.ABHC.REC.1397.076). All experimental animals were kept under normal humidity (50% ± 10%), temperature (24 ± 1 °C), and light (12 hr day/night cycle), with free access to water and rodent chow.

***Experimental design***

**Figure 1:**
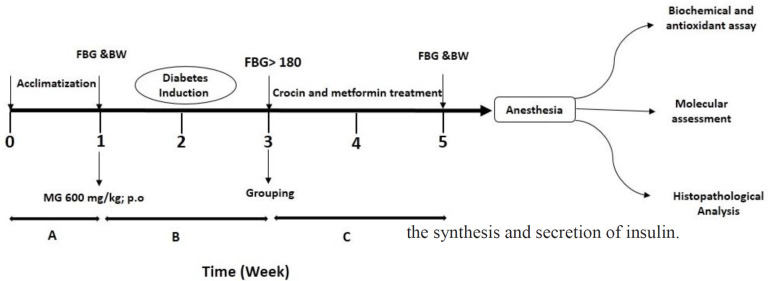
Diagrammatic representation of experimental protocol. A: Acclimatization lasted a week, B: Diabetes induction by MG (600 mg/kg), C: Administration of crocin (15, 30 and 60 mg/kg) and metformin (150 mg/kg). At the end of the 3rd week, diabetic mice were selected based on their FBG levels and divided into MG, MG+Crocin 15 mg/kg, MG+Crocin 30 mg/kg, MG‏+Crocin 60 mg/kg, and MG+metformin 150 mg/kg lasting 2 weeks. At the same time, the Control group received normal saline. Finally, after anesthesia, the following tests were evaluated

Induction of diabetes in animals was performed by daily MG (600 mg/kg. p.o.) administration (24) for 28 consecutive days (Figure 1). The mice were fasted for more than 6 hr, after 4 weeks MG administration to measure fasting blood glucose (FBG) from the tail vein. In this study, the mice with an FBG value of more than 180 mg/dl were considered diabetic mice (25). The animals were adapted for one week before the start of the experiment. After two weeks from the start of the experiment, concomitant administration of MG was treated with crocin (at three different doses) and metformin (positive control) as treated diabetic groups. Then, animals were randomly divided into six groups with ten animals in each group, which included the following grouping, respectively: 

CN (Control, received normal saline for 35 consecutive days)

MG (diabetic with MG)

MG + C15 (diabetic received crocin 15 mg/ kg)

MG + C30 (diabetic received crocin 30 mg/kg)

MG + C60 (diabetic received crocin 60 mg/kg)

MG + MT (diabetic received metformin 150 mg/kg as standard antidiabetic medication) (26).

At the end of the 4-week treatment with the above compounds, the animals were sacrificed by the combination of ketamine 10% + xylazine 2% (90 +10 mg/kg, respectively) to collect pancreas tissues using for enzymatic and molecular evaluation, and histological assay, so, tissues were washed with normal saline instantly, snap-frozen in liquid nitrogen, and reserved at -80 °C. Plasma samples were obtained by cardiac puncture blood collection and centrifuging at 3000 rpm for 15 min. All of the plasma samples were stored at -20 °C before use. 

***Measurement of MDA, anti-oxidant enzymes, GSH and Nrf2 levels, and GLO1 activity in pancreas homogenate***

The pancreas tissues were homogenized in ice-cold Tris–HCl buffer (0.1 M, pH 7.4) using the ratio (1:4 w/v), then centrifuged at 2000×g for 15 min at 4 °C. The supernatants were prepared and used to evaluate pancreas MDA, CAT, SOD, GSH, Nrf2 levels, and GLO1 activity.

***Diabetic variable and lipid profiles measurement***

The concentration of plasma insulin was measured by ELISA assay kits; Homeostatic Model Assessment for Insulin Resistance (HOMA-IR) and Homeostasis Model Assessment of β-cell Function (HOMA- β) were calculated by the following formula:

HOMA-IR: Insulin (µIU/ml) × FBG (mg/dl) / 405

HOMA-β: Insulin (µIU/ml) × 360 **/** [FBG (mg/dl) – 63] 

Quantitative insulin sensitivity check index (QUICKI) (27) and insulin disposition index (DI) were calculated by using the following formulas:

QUICKI: 1/ [log FBS (mg/dl) + log insulin (µIU/ml)] 

Insulin DI: HOMA-β/HOMA-IR

The plasma level of total cholesterol (TC), triglyceride (TG), High-density lipoprotein cholesterol (HDL-c), low-density lipoprotein cholesterol (LDL-c), and very-low-density lipoprotein cholesterol (VLDL–c) were assessed using commercial kits (Pars Azmoon, Iran) and the auto-analyzer method. 

***Isolation of microRNAs and cDNA synthesis***

Total microRNAs were isolated from the pancreas homogenate; using the High Pure miRNA Isolation kit (Roche, Germany) in accordance with the manufacturer’s protocol. At first, the concentration and purity of microRNA were determined by spectrophotometry at wavelengths 260 and 280 nm (Nanodrop Thermo Scientific S.N: D015), and then, cDNA was obtained from 1 µg of microRNA using the miScript II RT Kit (Qiagen, GmbH, Germany) according to the manufacturer’s manual.

***Quantitative real-time PCR (qRT-PCR)***

Quantitative real-time polymerase chain reaction (qRT-PCR) characterized microRNAs expression levels using a Light Cycler® 96 Real-Time PCR System (Roche Diagnostics, Indianapolis, IN, USA). All PCR amplifications were carried out in duplicate reactions in a final volume of 20 µl containing 2 µl cDNA, 10 µl 2x QuantiTect SYBR Green PCR Master Mix, 2 µl 10x miScript Primer Assay [miR-204 (MIMAT0000237), miR-216b (MIMAT0003729), miR-29a (MIMAT0004631), or miR-192 (MIMAT0000517), 2 µl 10x miScript Universal Primer [(MS0003374), (QIAGEN)], and 4 µl RNAase free water using the following protocol: First activation step at 95 °C for 15 min to activate HotStar Taq DNA Polymerase followed by 45 cycles at 94 °C for 15 sec, 55 °C for the 30 sec , and 70 °C for 30 sec . Also, H_2_O as a no-template negative control was routinely run in every PCR. MicroRNA relative expression was normalized to miR-16 as an internal control, and the transcript alterations were calculated using the 2−ΔΔCT method.

***Histological analysis***

For histological evaluation, isolated pancreas tissues were washed with normal saline and fixed in a 10% neutral formalin solution. After 72 hr of stabilization, in order to dehydrate tissues, they were placed in a series of graded alcohol, transferred to paraffin, and cut into 5 𝜇m sections by using a microtome (Leica RM 2125, Leica Microsystems Nussloch GmbH, Germany). The incised sections were stained with hematoxylin and eosin and used to assess the diameter of pancreatic islets by using a digital research microscope (BMZ-04- DZ, Behin Pajouhesh ENG. CO., Iran).

***Statistical analysis***

The data were presented as mean ± SEM and analyzed using GraphPad Prism 9 for Windows (GraphPad Software, San Diego, CA). For comparison among different groups, one-way analysis of variance (ANOVA) was used, followed by *post hoc* high significant difference (HSD) tests or chi-square test (with Bonferroni correction method). Statistical significance was set at *P*<0.05.

## Results

***Effects of crocin and MT on glycemic and insulin markers in MG-induced T2D***

**Figure 2:**
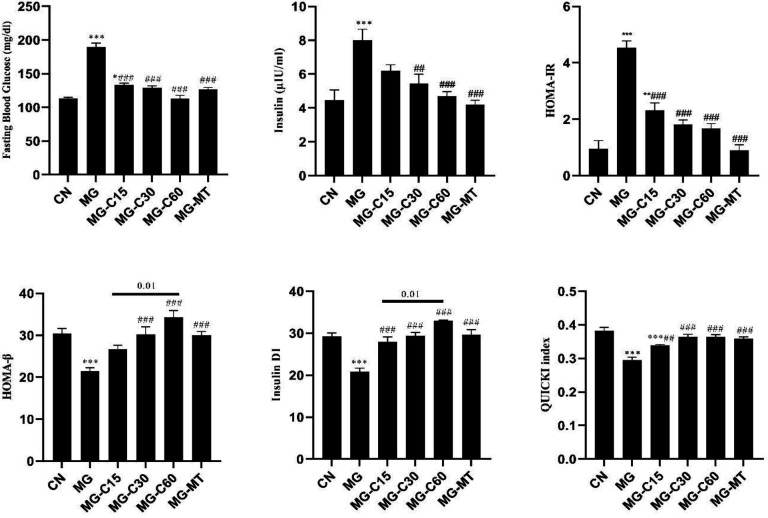
Effects of crocin and MT on glycemic and insulin markers in MG induced T2D

CN: control; MG: diabetic with methylglyoxal administration; MG‏-C15: diabetic‏+Crocin 15 mg/kg; MG-C30: diabetic‏+Crocin 30 mg/kg; MG-C60: diabetic+Crocin 60 mg/kg; MG-MT: diabetic+metformin 150 mg/kg. Data are presented as mean ± SEM, n=10 in each group. * Significant difference with CN; # Significant difference with MG; one symbol *P*<0.05; 2 symbols *P*<0.01; 3 symbols *P*<0.001

As shown in Figure 2, FBG was significantly higher in the MG group (*P*<0.001) compared with the CN group, and treatment with crocin and MT reduced FBG level as compared with the MG group (*P*<0.001). Moreover, insulin levels were higher in the MG group than in the CN group (*P*<0.001). Administration of crocin at 30 and 60 mg/kg and MT markedly reduced insulin levels. In addition, calculations of HOMA-IR, insulin DI, and QUICKI index were conducted to evaluate insulin resistance. It was indicated that insulin sensitivity decreased, as disclosed by increased HOMA-IR (*P*<0.001). Also, HOMA-β, insulin DI, and QUICKI values were reduced in the MG group (*P*<0.001) compared with the CN group. Treatment with crocin at 30 and 60 mg/kg and MT significantly increased HOMA-β values. Evaluation of HOMA-β indicated a significant difference between C15 and C60 groups (*P*<0.01). Insulin DI and QUICKI significantly increased in diabetic treated groups.

***Effects of crocin and MT on lipid profile in MG-induced T2D***

**Figure 3:**
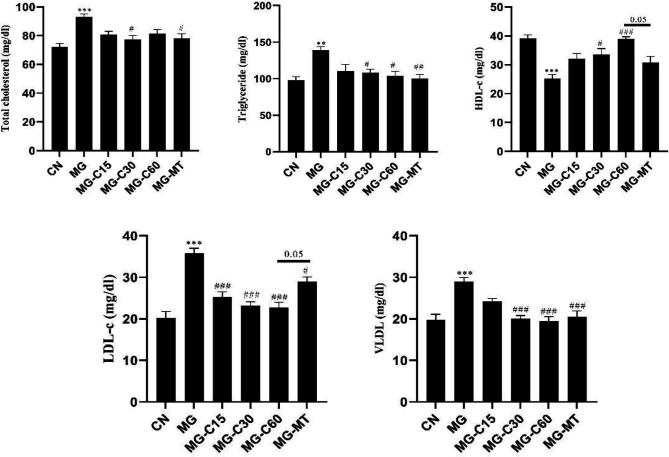
Effects of crocin and MT on lipid profile in MG induced T2D. CN: control; MG: diabetic with methylglyoxal administration; MG‏-C15: diabetic‏+Crocin 15 mg/kg; MG‏-C30: diabetic‏+Crocin 30 mg/kg; MG‏-C60: diabetic‏+Crocin 60 mg/kg; MG-‏MT: diabetic‏+metformin 150 mg/kg. Data are presented as mean ± SEM, n=10 in each group. * Significant difference with CN; # Significant difference with MG; one symbol *P*<0.05; 2 symbols *P*<0.01; 3 symbols *P*<0.001

The effects of crocin and MT on lipid profile were shown in Figure 3. The levels of cholesterol, triglycerides, LDL-c, and VLDL significantly increased in the MG group compared with the CN group. However, in evaluation of total cholesterol: crocin 30 mg/kg (*P*<0.05) and MT (*P*<0.05), triglycerides: crocin 30 and 60 mg/kg (*P*<0.05) and MT (*P*<0.01), LDL-c: all doses of crocin (*P*<0.001) and MT (*P*<0.05), VLDL: crocin 30 and 60 mg/kg and MT (*P*<0.001) significantly affected reduction of parameters mentioned when compared with the MG group. The effect of crocin 60 mg/kg on LDL-c level was better than MT (*P*<0.05). The MG group had a significant reduction in the plasma level of HDL-c compared with the CN group (*P*<0.001). Whereas, crocin (30 and 60 mg/kg) significantly increased plasma HDL-c in the MG treated mice. However, the effect of crocin 60 mg/kg on HDL-c was more than MT (*P*<0.05).

***Effects of crocin and MT on pancreatic oxidative stress biomarkers, GSH, GLO1, and Nrf2 levels in MG-induced T2D***

**Figure 4:**
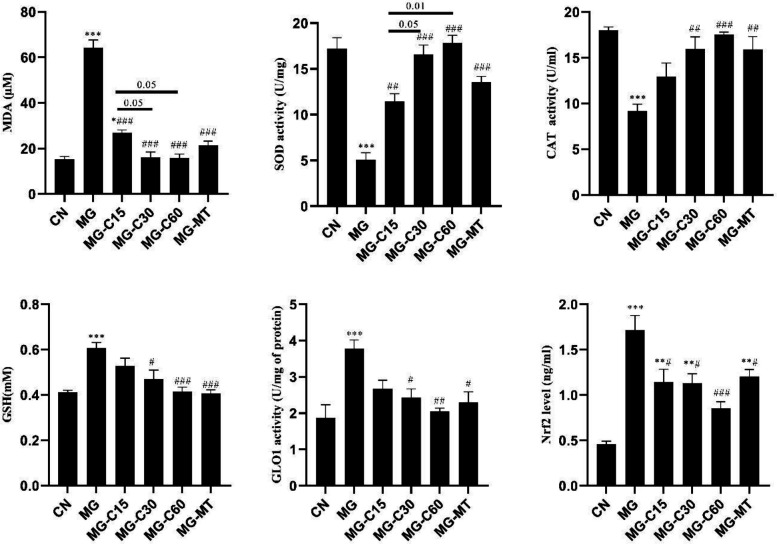
Effects of crocin and MT on pancreatic oxidative stress biomarkers, GSH, GLO1, and Nrf2 levels in MG induced T2D. CN: control; MG: diabetic with methylglyoxal administration; MG-C15: diabetic+Crocin 15 mg/kg; MG-C30: diabetic+Crocin 30 mg/kg; MG-C60: diabetic+Crocin 60 mg/kg; MG-MT: diabetic+metformin 150 mg/kg. Data are presented as mean ± SEM, n=10 in each group. * Significant difference with CN; # Significant difference with MG; one symbol P<0.05; 2 symbols *P*<0.01; 3 symbols *P*<0.001

Evaluation of oxidative and anti-oxidative markers indicated that the MDA level in the MG group was markedly higher than in the CN group, and treatment with crocin and MT significantly reduced it (Figure 4). Also, it was found that crocin at 30 and 60 mg/kg had better function than 15 mg/kg (*P*<0.05). Moreover, our results showed that MG reduced SOD and CAT activity in the MG group (*P*<0.001). However, crocin and MT therapy significantly improved these variables similar to the CN group. Crocin at the high dose was more effective than in the low dose. Furthermore, our results showed that GSH content significantly increased in the MG group. Administration of crocin (30 and 60 mg/kg) and MT significantly recovered GSH content in the MG-treated mice. But crocin 15 mg/kg had no significant effect on GSH content in diabetic mice. Also, we observed that GLO1 activity significantly increased in the MG group compared with the CN group (*P*<0.001), while treatment with crocin (30 and 60 mg/kg) and MT significantly decreased it similar to the control. Moreover, the increased level of Nrf2 in the MG group (*P*<0.001), significantly decreased in diabetic treated groups. The effect of crocin and MT groups on GLO1 activity and Nrf2 levels was similar (Figure 4).

***Effects of crocin and MT on pancreatic expression of miR-204, miR216-b, miR-192, and miR-29a in MG-induced T2D***

**Figure 5:**
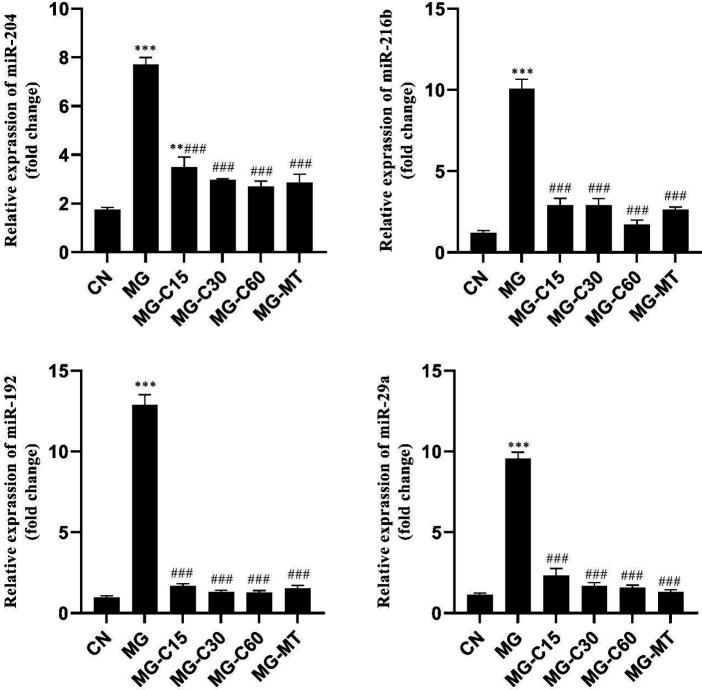
Effects of crocin and MT on the pancreatic expression of miR-204, miR-216b, miR-192, and miR-29a in MG induced T2D. CN: control; MG: diabetic with methylglyoxal administration; MG-C15: diabetic+Crocin 15 mg/kg; MG-C30: diabetic+Crocin 30 mg/kg; MG-C60: diabetic+Crocin 60 mg/kg; MG-MT: diabetic+metformin 150 mg/kg. Data are presented as mean ± SEM, n=10 in each group. * Significant difference with CN; # Significant difference with MG; one symbol *P*<0.05; 2 symbols *P*<0.01; 3 symbols *P*<0.001

The tissue-specific miRNAs profile results show that expression of miR-204, 216b, 192, and 29a increased in the MG group compared with the CN group (*P*<0.001). However, crocin and MT decreased expression of miR-204, 216b, 192, and 29a in all diabetic treated groups (*P*<0.001). In all treatment groups, crocin and MT had the same effects (Figure 5).

***Effects of crocin and MT on diameter and histology of pancreatic islets in MG-induced T2D***

**Figure 6:**
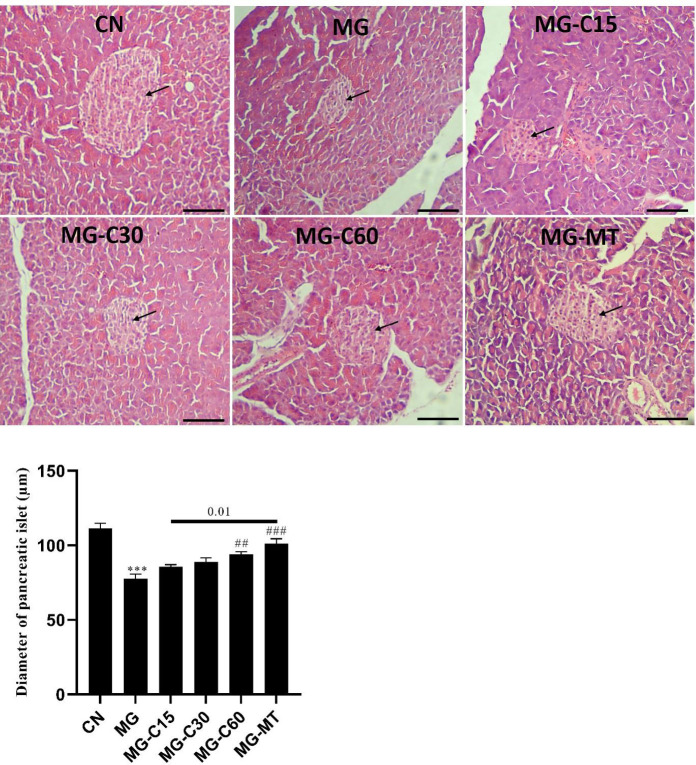
Effects of crocin and MT on diameter and histology of pancreatic islets in MG induced T2D. Scale bar: 100 µm. CN: control; MG: diabetic with methylglyoxal administration; MG-C15: diabetic+Crocin 15 mg/kg; MG-C30: diabetic+Crocin 30 mg/kg; MG-C60: diabetic+Crocin 60 mg/kg; MG-MT: diabetic+metformin 150 mg/kg. Data are presented as mean ± SEM, n=10 in each group. * Significant difference with CN; # Significant difference with MG; 2 symbols *P*<0.01; 3 symbols *P*<0.001

Pancreatic histology sections and islet diameter are represented in Figure 6. No lesion was observed in the CN group. A significant decrease was seen in β-islets diameter in the MG group compared with the CN group (*P*<0.001). Crocin 60 mg/kg and MT treated diabetic mice showed significantly increased β-islets diameter compared with the MG group (*P*<0.01 and *P*<0.001, respectively). Moreover, MT effectively improved β-islets diameter compared with crocin 15 mg/kg (*P*<0.01).

## Discussion

Accumulating evidence verify that MG induced insulin resistance by formation of AGEs. The current study demonstrated that MG induced T2D and insulin resistance, and this action was related to the enhancement of FBG and HOMA-IR and decreasing of HOMA-β, QUICKI, and insulin DI. Also, the pancreatic expression of miR-204, miR-216b, miR-192, and miR-29a increased and the levels of GSH, Nrf2, and GLO1 enhanced in the MG group. Crocin is an active component of saffron that has many biological effects. Our study showed that crocin attenuated diabetes-induced by MG. Also, Actually, Quantitative Real-Time PCR results also showed that crocin regulated miRNAs levels and reduced GLO1 enzymatic activity accompanied with regulating of GSH level, an integrant cofactor of GLO1 in the pancreas of diabetic mice.

It is widely known that MG can induce hyperglycemia and insulin resistance (28). In addition, hyperglycemia induces mitochondrial dysfunction and ER stress, promotes ROS generation, disturbs the anti-oxidant defense system, and finally causes cell apoptosis (29). Also, it was reported that there is a direct interaction between mitochondria, as a major source of ROS, and ER. So, overproduction of ROS in mitochondria during an overload of glucose leads to oxidative stress and subsequently promotes ER stress. Pancreatic β-cells seem to be especially susceptible to ER dysfunction because of the extensive distribution of ER and its functionality (7). Furthermore, the presence of large amounts of mitochondria in pancreatic β-cells, make them more susceptible to oxidative damage under high glucose conditions (30). In light of current descriptions, in our study, increased MDA level indicated an impaired mitochondrial function and, consequently, increased lipid peroxidation in pancreatic β-cells of the MG group. Also, our findings based on a reduction of SOD and CAT activity in mice that received MG alone imply that the balance of the anti-oxidant system is disturbed in hyperglycemic conditions. Administration of crocin especially at high dose markedly improved the levels of oxidant and anti-oxidant variables mentioned above. In line with our results, a report verified that MG increased oxidative stress by increasing MDA levels and decreased SOD and CAT activity in β-cells (31). 

Although, other enzymes such as MG reductase, aldo-ketoreductase, and aldehyde dehydrogenase have been shown to metabolize MG to lactaldehyde, hydroxyacetone, and pyruvate, respectively; the main enzyme for detoxifying cellular MG is GLO1 that converts MG into D-lactoylglutathione and D-lactate with a catalytic amount of GSH as a cofactor (32). Furthermore, a report verifies that Nrf2 acts as a positive regulator of MG detoxification by phase II enzymes in liver tissue (28). Our data revealed that Nrf2 and GLO1 activity was elevated in the MG group, while, crocin especially at high dose, and MT treatment exhibited normal Nrf2 levels and GLO1 activities in diabetic treated mice close to the control group. Moreover, increased amounts of GSH in MG-treated mice were alleviated through administration of crocin, at average and high doses, and MT in diabetic treated mice. So, it can be cocluded that ER stress improved through NrF2 activation and NrF2 stimulating effect on GSH and GLO1. According to the current conclusion, MG-dependent alteration in Nrf2 level and GLO1 activity was modified in groups with crocin treatment. In agreement with our results, it was reported that natural anti-oxidants prevent MG-induced glycative stress through activation of the Nrf2-GLO1 pathway (33).

Hyperglycemia occurs in the high level of plasma MG condition and leads to insulin resistance, adipocyte tissue dysfunction accompanied by vast dyslipidemia by enhanced VLDL in the fasting state, and intestinal chylomicron making in postprandial condition (34). Also, diabetic dyslipidemia caused enhancement of triglycerides, LDL, and decreased HDL cholesterol levels (35). So, it is clear that maintaining FBG close to the optimum level and modifying insulin biomarkers, prevents disruption of the lipid profile and can be useful in the treatment of diabetes. Our findings revealed that crocin treatment was effective in alleviating FBG levels and insulin homeostasis and thus improved glycemic control under the high MG conditions. Furthermore, crocin markedly improved the lipid profile changes of MG-treated mice. Our findings are in agreement with previous studies, in which, crocin effectively improved diabetic symptoms such as FBG level, lipid profile disturbance, and insulin sensitivity in diabetic animals (36).

MicroRNAs importantly affect the β-cell function and act as mediate regulators of insulin resistance. They can also be as predictable small molecules of diabetes progression. In our study, miR-204, 216b, 192, and 29a expressions were evaluated to determine molecular alterations in pancreas tissue. As previously reported, miR-204 is distinguished as an important modulator of ER stress in pancreatic β-cell (13). In addition, miR-216b specifically has been determined for acute pancreatic cell injury (14). MiR-192 is abundantly elevated in a prediabetic state and suppresses proliferation and regulation of pancreatic β-cells and insulin secretion (15). Also, in response to the high glucose condition, miR-29a is up-regulated in β-cells and modulates β-cell dysfunction and its proliferation, while suppression of miR-29a improves insulin secretion (37). According to a previous study, overexpression of miR-204 can indirectly alter the Nrf2 level (38). Another study reported that miR-204 is an exact indicator of ROS-associated ER stress (39). So, the levels of Nrf2 may be affected by miR-204 alterations during ER stress. These findings are in agreement with our results, in which, miR-204 changes are consistent with Nrf2 changes in the MG group. Furthermore, it was reported that ER stress causes the levels of miR-216b to multiply several times (40). In our study, increased expression levels of miR-204 and miR-216b in the MG group indicated that ER stress and cell damages clearly occurred in the MG group; crocin and MT administration alleviated them. Moreover, miR-192 overexpression in the MG group inhibited β-cell proliferation and insulin secretion that was restored by using crocin and MT treatment. The current result was confirmed by histology evaluation, at which, β-cell proliferation and diameter of Langerhans islets were reduced in the MG group. Also, an increased amount of miR-29a expression in the MG group indicated an impairment of β-cell dysfunction**, **and a decreased amount of miR-29a expression in diabetic treated mice indicated an improvement in β-cells function.

## Conclusion

Oxidative stress can lead to ER stress, both of which occur as a result of excessive ROS production. The pancreas is more prone to ER stress damage due to its extensive endoplasmic reticulum. Damage to the endoplasmic reticulum will lead to impaired insulin production and eventually to diabetes. Our results indicated that daily administration of MG impaired pancreatic β-cells function. Among the microRNAs, miR-204 and 216b were used to diagnose ER stress. Flavonoids have been widely used in the treatment of various diseases, including diabetes. In this study, crocin modulated the anti-oxidant defense system by reducing ROS production and lipid peroxidation. Also, crocin played an important role in the regulation of Nrf2 and GLO1 levels in MG-receiving mice through reducing oxidative stress and ultimately ER stress. This finding was confirmed by observing the effectiveness of crocin in modulating ER stress-related microRNAs. These results will be helpful to a better understanding of the exact mechanism of crocin treatment against high MG conditions. 

## Authors’ Contributions

AA and VR Conceived and designed the project; AA, VR, SAM, and LK Performed data processing and collection and performed experiments; AA Analyzed and interpretated the results; AA and VR Prepared the draft manuscript and visualization; VR Critically revised and edited the article; AA, VR, SAM, and LK Approved the final version to be published; AA Provided supervision and funding acquisition.

## Conflicts of Interest

 There are no conflicts of interest, and all authors support the submission to this journal.
